# Mixed methods evaluation to explore participant experiences of a pilot randomized trial to facilitate self‐management of people living with stroke: Inspiring virtual enabled resources following vascular events (iVERVE)

**DOI:** 10.1111/hex.13584

**Published:** 2022-08-23

**Authors:** Tara Purvis, Doreen Busingye, Nadine E. Andrew, Monique F. Kilkenny, Amanda G. Thrift, Jonathan C. Li, Jan Cameron, Vincent Thijs, Maree L. Hackett, Ian Kneebone, Natasha A. Lannin, Dominique A. Cadilhac

**Affiliations:** ^1^ Stroke and Ageing Research, Department of Medicine, School of Clinical Sciences at Monash Health Monash University Clayton Victoria Australia; ^2^ Population Health Australian Institute of Health and Welfare Canberra ACT Australia; ^3^ Peninsula Clinical School, Central Clinical School Monash University Melbourne Victoria Australia; ^4^ National Centre for Healthy Ageing Monash University Melbourne Victoria Australia; ^5^ Stroke Division, Florey Institute of Neuroscience and Mental Health The University of Melbourne Heidelberg Victoria Australia; ^6^ Department of Electrical and Computer Systems Engineering Monash University Melbourne Victoria Australia; ^7^ Australian Centre for Heart Health Royal Melbourne Hospital Melbourne Victoria Australia; ^8^ Department of Neurology Austin Health Heidelberg Victoria Australia; ^9^ The George Institute for Global Health University of New South Wales Sydney New South Wales Australia; ^10^ Faculty of Health and Wellbeing University of Central Lancashire Preston Lancashire United Kingdom; ^11^ Graduate School of Health University of Technology Sydney Ultimo New South Wales Australia; ^12^ Department of Neuroscience Monash University Melbourne Victoria Australia; ^13^ Allied Health Directorate Alfred Health Melbourne Victoria Australia

**Keywords:** digital health, eHealth, feedback, goal setting, participant experience, self‐management, stroke

## Abstract

**Introduction:**

Despite digital health tools being popular for supporting self‐management of chronic diseases, little research has been undertaken on stroke. We developed and pilot tested, using a randomized controlled design, a multicomponent digital health programme, known as Inspiring Virtual Enabled Resources following Vascular Events (iVERVE), to improve self‐management after stroke. The 4‐week trial incorporated facilitated person‐centred goal setting, with those in the intervention group receiving electronic messages aligned to their goals, versus limited administrative messages for the control group. In this paper, we describe the participant experience of the various components involved with the iVERVE trial.

**Methods:**

Mixed method design: satisfaction surveys (control and intervention) and a focus group interview (purposively selected intervention participants). Experiences relating to goal setting and overall trial satisfaction were obtained from intervention and control participants, with feedback on the electronic message component from intervention participants. Inductive thematic analysis was used for interview data and open‐text responses, and closed questions were summarized descriptively. Triangulation of data allowed participants' perceptions to be explored in depth.

**Results:**

Overall, 27/54 trial participants completed the survey (13 intervention: 52%; 14 control: 48%); and 5/8 invited participants in the intervention group attended the focus group. *Goal setting*: The approach was considered comprehensive, with the involvement of health professionals in the process helpful in developing realistic, meaningful and person‐centred goals. *Electronic messages (intervention)*: Messages were perceived as easy to understand (92%), and the frequency of receipt was considered appropriate (11/13 survey; 4/5 focus group). The content of messages was considered motivational (62%) and assisted participants to achieve their goals (77%). Some participants described the benefits of receiving messages as a ‘reminder’ to act. *Overall trial satisfaction*: Messages were acceptable for educating about stroke (77%). Having options for short message services or email to receive messages was considered important. Feedback on the length of the intervention related to specific goals, and benefits of receiving the programme earlier after stroke was expressed.

**Conclusion:**

The participant experience has indicated acceptance and utility of iVERVE. Feedback from this evaluation is invaluable to inform refinements to future Phase II and III trials, and wider research in the field.

**Patient or Public Contribution:**

Two consumer representatives sourced from the Stroke Foundation (Australia) actively contributed to the design of the iVERVE programme. In this study, participant experiences directly contributed to the further development of the iVERVE intervention and future trial design.

## 1 INTRODUCTION

Advances in stroke treatments have reduced mortality, resulting in more people living with a disability.^1^ Consequently, there has been a growing prevalence of people living with long‐term physical, psychological and social effects of stroke.^2^ Many people living with stroke report unmet needs and ongoing problems with fatigue, cognition, concentration, and emotions up to 2 years poststroke.^3^ Self‐management encourages ‘active participation’ of people in their own health recovery, to reduce the overall physical, emotional and psychosocial impact of their illness.^4^, ^5^ While self‐management encompasses a broad range of approaches, the process typically includes aspects of problem solving, goal setting, using available resources, making choices and taking action to assist people to acquire the knowledge, confidence and skills to manage their condition.^6^ For people living with stroke, there is emerging evidence indicating that self‐management programmes may be of benefit, particularly related to improvements in quality of life and self‐efficacy.^6^, ^7^ Goal setting accompanied by methods of support has been shown to facilitate self‐management.^8^

Numerous generic digital health self‐management tools exist, and commonly include mobile technology applications (mHealth), internet‐based programmes, use of short message services (SMS) or email.^9^ These tools have been trialled with positive outcomes in various populations to improve disease self‐management, lifestyle behaviours or achieve health goals.^10^, ^11^, ^12^ However, there is limited information on the application in people with stroke. Consequently, we developed a multicomponent digital health programme, Inspiring Virtual Enabled Resources following Vascular Events (iVERVE), to improve self‐management after stroke, designed with input from consumers, researchers and clinicians. The iVERVE programme incorporated a facilitated approach to set person‐centred recovery and health‐related goals,^13^ utilizing a menu‐based template. After goals were set, those in the intervention group received electronic messages (SMS or email) aligned with their expressed goals, while the control group received limited administrative messages only.^14^ The feasibility and potential effectiveness of the iVERVE programme was tested in a 4‐week Phase I, prospective randomized controlled trial (RCT) in people 12–14 months poststroke.^15^ Potential for improvement was identified in elements of self‐management (Health Education Impact Questionnaire) and several health‐related quality of life domains (EQ‐5D‐3L).^15^

Given this was a new programme designed for this population group, it was important to obtain feedback on acceptability, including participant satisfaction, with the various components of the iVERVE programme, as part of the process evaluation. In this paper, we aimed to explore the participant experience and perceptions of the multicomponent iVERVE programme, to further inform future Phase II and III trials of the programme, in addition to the design of future digital health trials.

## 2 METHODS AND MATERIALS

### 2.1 Study design and participants

Mixed methods study, involving satisfaction surveys and focus group interviews of participants in the iVERVE trial (March 2017 to September 2017). Participants were people with stroke living in the community in Victoria, Australia, recruited from the Australian Stroke Clinical Registry.^16^ Reporting of this study is underpinned by the Survey Reporting Guideline (SURGE),^17^ and the COnsolidated criteria for REporting Qualitative research (COREQ).^18^

### 2.2 Description of the iVERVE trial

**Figure 1:**
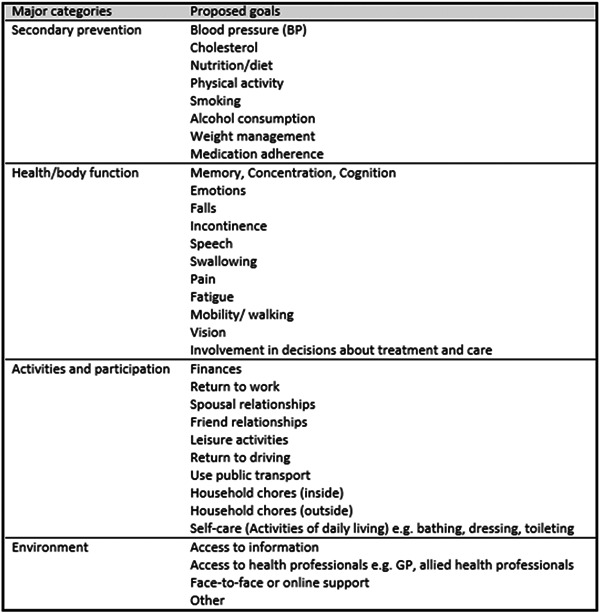
Initial iVERVE goal setting menu template. iVERVE, Inspiring Virtual Enabled Resources following Vascular Events. Reproduced from the development phase of this project (Patient Preference and Adherence), under the Creative Commons Attribution License (https://creativecommons.org/licenses/by/4.0/). An adaptation was made to include ‘other’ as an option under the environment category.

A detailed description of the iVERVE trial has been published.^15^ In brief, a standardized process for goal setting was undertaken to develop goals that were important and meaningful to the participant, and that would most likely be attainable within the 4‐week intervention timeframe, as well as measurable (i.e., SMART goals; specific, measurable, attainable/achievable, relevant and timely).^19^ The process involved using an open‐ended menu‐based template as a prompt to encourage participants to set goals across broad areas related to recovery or health management after stroke. The participants were encouraged then to identify up to three goals that were important to them. The process was facilitated by a health professional, who fostered reflection on what was meaningful to the participant and supported the participant to come up with the measurable aspects related to the goal (SMART criteria), rather than influencing the types of goals that were set. The menu covered areas of secondary prevention, health and body function, activities and environment mapped to the International Classification of Function domains with the addition of a secondary prevention category (Figure 1).^14^ Therefore, participants formulated person‐centred recovery or health goals based on the broad themes of the menu but were not restricted to these categories and subcategories.

**Figure 2:**
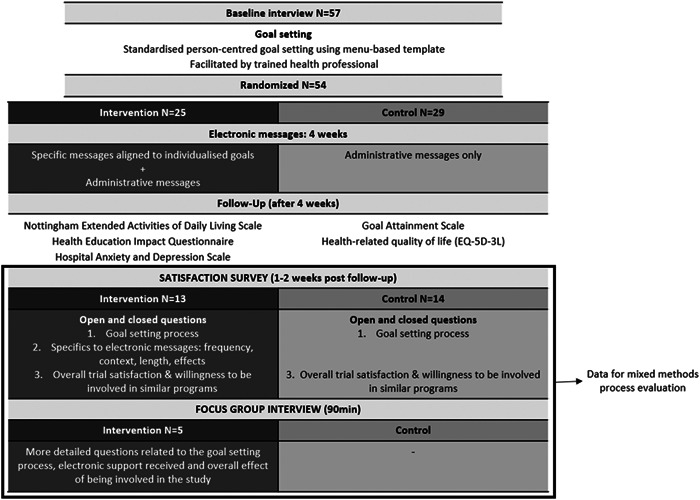
Outline of the iVERVE trial and data collected for the process evaluation. iVERVE, Inspiring Virtual Enabled Resources following Vascular Events.

After participants set their goals, those randomized to the intervention group received electronic support messages aligned to their goals. The frequency of the messages received varied based on the number of goals set; each week participants received an average of two messages per goal in addition to a general motivational message and the standard administrative messages (e.g., welcome message and reminder message about the time of follow‐up assessment). The control group received 2–3 administrative messages only during the 4‐week postrandomization (Figure 2). The primary outcomes were study retention, goal attainment^20^ and satisfaction, with secondary outcomes, including self‐management,^21^ emotional status,^22^ health‐related quality of life^23^ and participation,^24^ measured 1–2 weeks post the programme delivery. Relevant licences/permission were obtained for tools used in the outcome assessments (hei‐Q—https://eprovide.mapi-trust.org/instruments/health-education-impact-questionnaire and EQ‐5D‐3L—https://euroqol.org/publications/user-guides/).

### 2.3 Data collection

Data from the satisfaction surveys (control and intervention) and the focus group interview (intervention group only) were used for this study (Figure 2).

#### 2.3.1 Survey

One to two weeks post completion of the 4‐week trial, all participants (intervention and control) were mailed a satisfaction survey (including a reply‐paid envelope for return). The surveys, which were developed and adapted from published works,^25^, ^26^, ^27^, ^28^ included closed questions (*n* = 11) incorporating a 5‐point Likert scale (*strongly agree*, *agree*, *neutral*, *disagree*, *strongly disagree*) to elicit experiences and perceptions of the goal setting process, overall trial satisfaction and willingness to be involved with similar programmes. Additional open‐ended questions (*n* = 4) gave participants the opportunity to provide more in‐depth responses. Those in the intervention group were asked additional questions (*n* = 29 closed; *n* = 3 open) related to specifics of the electronic support messages, including frequency, context, effect and time period over which messages were received (Supporting Information: File SA). Participation in the survey was voluntary and consent was implied by completion.

#### 2.3.2 Focus group interview

A purposively selected sample of participants from the intervention group who responded to the survey, and who lived within 50 km of Monash University (Victoria), were mailed an invitation to participate in a focus group (*N* = 8). The sample was stratified to ensure the inclusion of participants with positive and negative views of the programme. The semi‐structured focus group schedule included a series of open‐ended questions to allow more in‐depth, rich data to be obtained related to the goal‐setting process, electronic support received and overall effect of being involved in the study (Supporting Information: File SB). To commence, participants were advised that their responses would be used to improve the intervention and that their perspective was important, regardless of whether it was positive or negative. Author T. P. (female, experienced qualitative researcher with MSci and expertize in stroke research and clinical care) conducted the focus group interview in person at Monash University (Melbourne) in September 2017. T. P. was also involved in the goal‐setting process with some participants. However, participants remained unaware of T. P.'s involvement in goal setting at the time of the focus group interview, and the allocation of individual IDs meant participants were unable to be readily identified by researchers involved in the interview. A second researcher was present at the focus group interview to record notes and nonverbal interactions between participants. The focus group interview was audio‐recorded and lasted approximately 90 min.

### 2.4 Data analysis

Data management software (NVivo 10; QRS International Pty Ltd.) was used to manage the qualitative data. Inductive thematic analysis (T. P.) was used with transcribed interviews. A coding tree outlining the major themes and subthemes was developed and used to systematically code responses.^29^ Coding was verified independently by another researcher (D. A. C., female, research PI, PhD, Public Health) to ensure the interpretation and meaning were maintained.

Open‐text responses from the surveys were analysed using a similar thematic approach. Descriptive statistics were used to summarize closed‐ended questions using Stata/SE 15.01 (StataCorp 2017). Missing responses to questions are depicted in the denominators presented in the results. Using triangulation, which involved comparing and contrasting the data collected from the multiple methods (surveys and interviews),^30^ a more complete exploration and understanding of the participant's perceptions of the iVERVE programme was able to be described. Analysis of the survey and focus group data occurred independently of, and before, the main results of the feasibility pilot RCT were known. Illustrative quotes are provided.

## 3 ETHICS

Ethics approval to develop and conduct the feasibility assessment of the iVERVE message system was provided by Monash University Human Research Ethics Committee (CF16/1920‐2016000979), with the trial retrospectively registered with the Australian New Zealand Clinical Trials Registry (ACTRN12618001519246). Participants provided written informed consent to participate in the trial, and focus group interview.

## 4 RESULTS

Of the 54 participants randomized, 27 completed the satisfaction survey (13 intervention: 52%; 14 control: 48%). Five of the eight invited (intervention group) participated in the focus group (Table 1). Characteristics of survey nonresponders were similar to responders (Supporting Information: File SC). Results are presented in chronological order related to the components of the iVERVE trial, with a summary of the suggested areas for improvement.

**Table 1:** Characteristics of participants who completed the satisfaction survey and participated in the focus group interview

	Satisfaction survey		Focus group *n*/*N* (%), *N* = 5
	Control *n*/*N* (%), *N* = 14	Intervention *n*/*N* (%), *N* = 13	
Sex, male	9/14 (64)	7/13 (54)	4/5 (80)
Age, median (Q1, Q3)	68 (57, 76)	70 (66, 79)	68 (67, 73)
Married, with partner	10/14 (71)	5/13 (38)	2/5 (40)
Lived alone	3/13 (23)	6/13 (46)	3/5 (60)
Lived home or unit	13/13 (100)	12/12 (100)	5/5 (100)
Employment status			
Employed/volunteer^a^	5/13 (38)	4/12 (33)	1/4 (25)
Unemployed	–	–	–
Retired	8/13 (62)	8/12 (67)	3/4 (75)
Preference for electronic messages			
SMS	5/14 (36)	7/13 (54)	3/5 (60)
Email	9/14 (64)	6/13 (46)	2/5 (40)
Number of goals set, median (Q1, Q3)	2 (2, 3)	2 (1, 3)	3 (2,3)
Major categories of goals set^b^			
Secondary prevention	10/32 (31)	9/27 (33)	5/12 (42)
Health/body function	12/32 (38)	5/27 (19)	2/12 (17)
Activities and participation	7/32 (22)	9/27 (33)	3/12 (25)
Environment	3/32 (9)	4/27 (15)	2/12 (17)

Abbreviations: Q1, first quartile; Q3, third quartile; SMS, short message service.

^a^ Full, part‐time or casual employment, or volunteer work.

^b^ With rounding, may not add to 100%.

### 4.1 Goal‐setting process (control and intervention)

**Figure 3:**
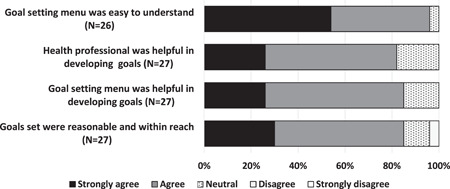
Satisfaction with the goal‐setting process (control and intervention groups).

The goal‐setting menu and assistance provided by the health professional were considered important for goal development (Figure 3). For each category within the goal setting menu, examples of the participant stated goals and the final SMART goals formulated have been included in Supporting Information: File SD.

Health professionals' input into the goal‐setting process appeared particularly important for setting realistic and meaningful goals, especially for those participants who may have suffered a milder stroke and were now independent.

[I felt like] *I was on track… but certainly someone ringing up was a help to maybe consider things that we hadn't looked at*. (Focus group: Participant 103, male, 66 years)

Even with input from the health professional, the importance of all goals being person‐centred was highlighted:

*I felt the goals were individualised and that I had ownership of the goals*. (Focus group: Participant 276, male, 67 years)

Focus group participants described the health professionals involved in the goal‐setting process as ‘….caring and understanding’. It was felt they were ‘…helpful’, ‘….encouraging’ and ‘…sensitive enough to prod, but not to push’ (focus group: Participant 16, female, 63 years). Overall, health professional invovlement was considered one of the most important aspects of participating in the iVERVE programme.

Overall, 96% of survey respondents felt the goal‐setting menu was easy to understand, provided a useful way to reflect on what was important to them and guided the process of developing goals:

….[using the goal setting menu] *made me consider what I was like before my stroke and really think about what I wanted to do, and what I was interested in….not something I had thought about doing prior to this project*. (Focus group: Participant 276, male, 67 years)

Survey respondents reported the goal‐setting menu covered all goals they wanted to address. Focus group participants noted that more recognition of the importance of goals about ‘communication’ and ‘emotional challenges related to living alone’ would be beneficial.

### 4.2 Electronic support messages (intervention only)

The use of digital health mediums was viewed as acceptable, with approximately two‐thirds of survey respondents reporting that having messages sent by SMS or email was a good way to educate people about stroke.

#### 4.2.1 Intervention group perceptions on the delivery of electronic support messages

The importance of using SMS or email to deliver health messages was evident. In general, the preference was based on prior familiarity with the electronic device (mobile phone, tablet or computer) and ease of access.

*I was familiar with email, and used to it, more so than text*. (Focus group: Participant 78, male, 73 years)

Very few technical issues were reported, with only one focus group respondent (Participant 103, male, 66 years) reporting that a ‘couple of emails ended up in my spam box …some did, some didn't’. While all survey respondents felt the messages were sent at an appropriate time of day, almost half (6/13) preferred messages to be sent in the morning.

#### 4.2.2 Frequency of messages sent and duration from intervention group participants

Participants were unaware that the number of messages received related to the number of goals set. From the survey, 85% of the 13 respondents (intervention) considered the frequency of the messages to be appropriate, which was similar in proportion to the focus group respondents. Nevertheless, there were occasional reports of the messages being ‘…too frequent’ and ‘more of an annoyance’, while others stated they like the repeated ‘reminder’. Although 12/13 survey respondents believed the 4‐week duration of messages was satisfactory, numerous focus group participants reported they were still working to achieve some identified goals. It was suggested that the duration of messages could be influenced by the type of goals set, and having a longer period of messaging would be more appropriate if the goal was around maintenance or long‐term lifestyle change.

*For some of us the goals were more long term…it was not just a goal that in 4 weeks you were done….you have to keep on exercising and keep on managing your weight*. (Focus group: Participant 103, male, 66 years)

#### 4.2.3 Perceptions from intervention group participants on the content of electronic support messages

Overall, 92% of survey respondents felt the SMS/email messages were easy to understand. However, only about one in two respondents reported that they understood how to access further information from the weblinks provided within the messages.

**Figure 4:**
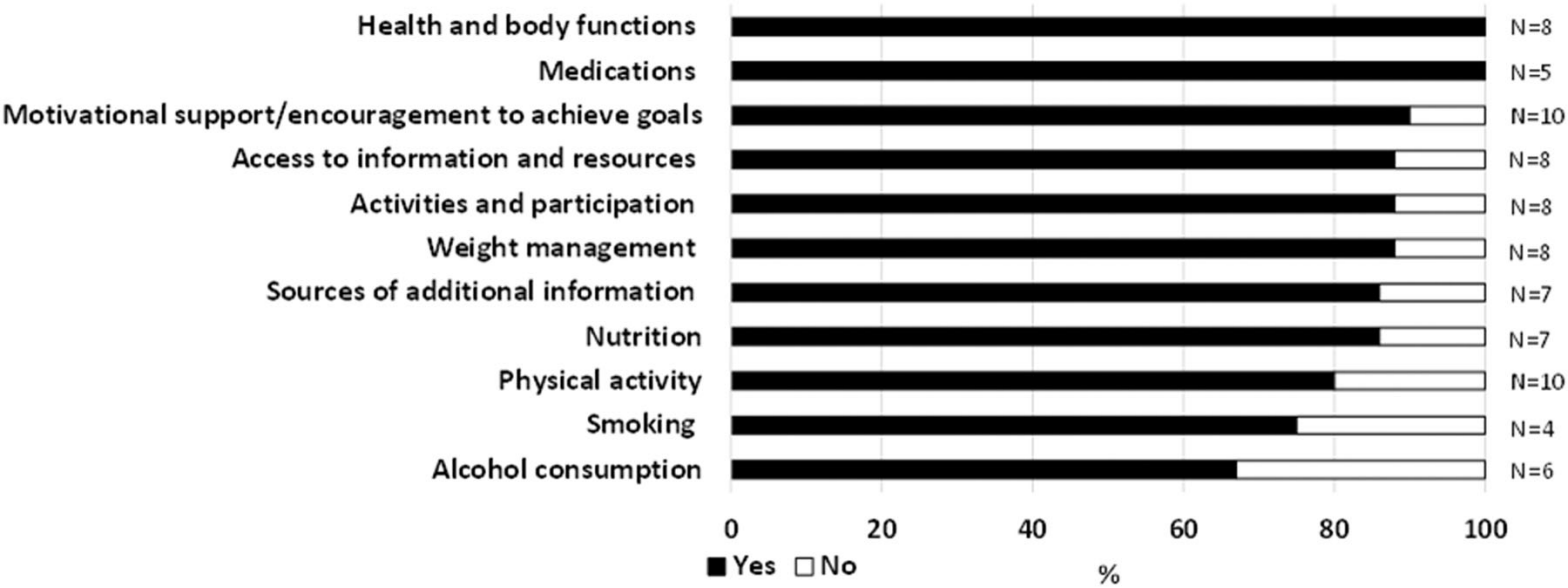
Proportion of intervention group participants who ‘liked reading’ messages related to specific goals. N = number of intervention group participants who received messages related to specific goal categories.

The electronic messages that were more positively received by intervention participants were those that provided support and encouragement to achieve the goals set (e.g., motivational), or referred to health and body functions (e.g., mobility, fatigue, falls, pain, emotions), or medications. The least applicable to the group were those related to smoking, and alcohol consumption (Figure 4).

Generally, the content of the messages was considered motivational (62%) and supportive, and assisted participants to achieve their goals (77%).

*It was just a little gentle reminder, I didn't find them intrusive…I would have a quick look…. they were easy to understand, relevant*, [the] *language was appropriate….I got the message, a quick reminder…it got me focused on the goals*. (Focus group: Participant 97, male, 80 years)

However, there were a few participants who described the messages as ‘…too general’ and ‘…impersonal’, with 3/13 (survey respondents) reporting that they stopped specifically reading all the content of the messages received. Even in these circumstances, respondents still believed the messages were beneficial, potentially as ‘reminders’ or prompts, rather than the full content of the messages.

*I would get a ‘ping’ to notify me I was getting an SMS, I wouldn't always read it, but I would think, ok come on I need to go for a walk, and take the dog out… so it was more of a guilt thing. On the whole it worked*…. (Focus group: Participant 103, male, 66 years)

### 4.3 Overall satisfaction and perceived benefit of involvement in the iVERVE trial (control and intervention)

In total, 81% of all survey respondents indicated that they would recommend participation in the iVERVE trial to other people with stroke (intervention: 85%; control: 79%), with the information provided perceived to be trustworthy (overall: 93%; intervention: 100%; control: 86%) and relevant (overall: 78%; intervention: 77%; control: 79%).

….[the project] *was quite good and relevant to my situation*. (Survey: control, Participant 309, male, 76 years)

A number of respondents (survey and focus group) expressed that a similar programme would have been useful earlier in their recovery. Although the recommended timing varied, it was generally agreed by four of the five focus group participants that within the first six months of stroke would be the most beneficial time period to start this programme.

*I feel it could have been suggested to me earlier on my recovery. Maybe on completion of my rehab*[ilitation]. (Survey: control: Participant 133, female, 73 years)

*I think earlier would have been better…you have a stroke, and realise you are not bullet proof….I remember reading through a little booklet* [My Stroke Journey] *when I had my stroke…but something like this would have helped me earlier*. (Focus group: Participant 276, male, 67 years)

Of those participating in the intervention component who received electronic messages related to their goals, 69% reported increased health awareness around individual health needs, and 85% felt their self‐management skills had improved. Almost two in five (38%) also described an increase in confidence in using electronic devices to find health information since completing the programme. Focus group participants all perceived that they had benefitted from being involved in the iVERVE trial. Ongoing advantages from the messages, including a continued focus on achieving their goals, occurred even after the messages ceased. Interestingly, several focus group respondents stated they had already set goals on some level before recruitment into the iVERVE trial. In some instances, this involved personal goals around going on a holiday in the future, lifestyle choices or physical activity. Regardless, all believed that the iVERVE trial was appropriate and important to facilitating goal attainment, and overall, was motivational and beneficial.

### 4.4 Summary of feedback on how to improve the programme (control and intervention)

**Figure 5:**
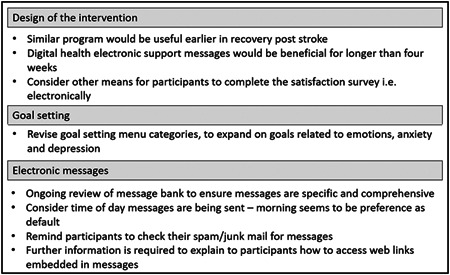
Overall feedback about participant experiences of the iVERVE trial. iVERVE, Inspiring Virtual Enabled Resources following Vascular Events.

Important feedback from participants, in both the control and intervention groups, on the overall iVERVE trial was obtained, a summary of which is provided in Figure 5.

## 5 DISCUSSION

We have described participant experiences, including acceptance, satisfaction and areas for improvement of people with stroke who participated in our Phase I RCT of the iVERVE trial. Overall, we have been reassured that the iVERVE programme, which includes person‐centred goal setting and electronic support messages aligned to goals, was acceptable and relevant. It was perceived that participating in the intervention component positively influenced health awareness around individual needs, improved self‐management skills and assisted participants to achieve their goals.

The use of digital health, including mobile applications and electronic health technology/interventions is becoming more popular. Its popularity is based on the potential to maximize equitable access to healthcare resources, minimize expenses to users and promote user flexibility and individualized care.^31^ People with stroke have reported interest in the use of technology for health information and self‐management,^32^, ^33^ indicating a promising potential alternative to more traditional means of face‐to‐face education and care provision. The focus on using technology in stroke is further emphasized by a number of published protocols integrating text messages, emails or use of applications into interventions to increase physical activity and improve diet,^34^ and focus on stroke prevention and self‐management ability,^35^ or reduce poststroke depression.^36^

There are few studies with interventions that have included person‐centred goal setting with aligned digital health support. In one study, goal setting was used to modify behaviours, as well as a weekly SMS, as part of a multifaceted intervention to improve control of blood pressure in patients with stroke.^11^ The SMS included general reminders regarding clinic visits, recommendations for lifestyle modifications, medication adherence and health beliefs. This intervention differed from iVERVE, where the goals cover all the diverse impacts of stroke including recovery, and the messages were aligned to individualized goals. Despite these differences, the prior investigations resulted in greater improvements in health behaviours in the intervention group for physical activity, nutrition and medication adherence than in controls.^11^ In people with stroke, the use of SMS has also been incorporated into interventions to improve walking performance and lower leg strength,^37^ and medication adherence.^38^

An essential component of digital health programmes is ensuring they are appropriately designed and targeted to users by taking into account the effects of respective health conditions.^39^ Consequently, there is growing recognition of the benefits of a codesign approach to ensure that the research questions, interventions and outcomes are relevant to clinicians, patients and other users.^40^, ^41^ The iVERVE trial was developed with input from researchers, clinicians, people living with stroke and advocates from the Stroke Foundation.^14^ However, further understanding of the acceptability and feedback related to the programme from participants supports translation and future work in the field to ensure that the needs of people living with stroke are met. This is particularly important considering the heterogeneity in digital health interventions and outcomes reported.^42^ Specifically, people living with stroke, are generally an older population. Many have limited experience of using electronic devices and may have reduced physical and cognitive function;^43^ factors that may affect access and use of mobile devices, computers and email.

Our results highlight the central role that health professionals had in facilitating the development of realistic, meaningful and person‐centred goals important to the participant. Being ‘caring and understanding’, ‘helpful’ and ‘encouraging’, aligns with the altruistic value often considered important to health professionals, which can influence their approach to person‐centred practice.^44^ Indeed, prior research has indicated there may be a mismatch between health professionals' perceptions of person‐centred practice and the reality, particularly in the rehabilitation setting,^13^, ^45^ Further, participants' own values of health professionals in the study may potentially have increased their engagement and motivation to work towards achieving outcomes. A few participants also reported that their behaviour was driven by a sense of guilt if they did not work to achieve their predetermined goals.

Offering SMS or email options for the distribution of the health messages was important, and the preference often aligned with those familiar to the participant.^42^ While two‐thirds of participants from the intervention group (survey) believed the message content was motivational, an additional benefit from the regular ‘reminder’ or prompt, may have also supported behaviour change.^11^, ^46^ Mornings were generally the preference to receive messages, and overall, 85% of participants were satisfied with the frequency of messages sent. Variation exists in the research related to the recommended frequency of messages to encourage behaviour change.^47^ While it is common for daily messages to be sent,^47^, ^48^ frequency can be influenced by the health behaviour being targeted,^47^, ^49^ the length of the intervention,^50^ with the ultimate aim to not overwhelm participants.

The specific feedback from participants related to their experience during this pilot study has reiterated the importance of testing the effectiveness of the self‐management support programme earlier in the stroke recovery continuum, and over a longer period. We have used this feedback to modify the protocol for our fully powered RCT,^51^ currently being undertaken within Australia. The Phase II and III trials are designed to provide the intervention for 12 weeks commencing 7–14 days after discharge from an acute care hospital. The aim is to test the effectiveness of the comprehensive electronic self‐management support programme on emergency department presentations/hospital readmissions, goal attainment, self‐efficacy, cardiovascular events, quality of life and costs. The intervention comprises a revised 34‐item goal‐setting menu, which includes more specific categories related to emotional challenges poststroke, an area highlighted by respondents as lacking in the original version (Phase I). The revised goal‐setting menu is supported by a newly developed 120‐page manual containing: guideline summaries; common goals; goal metrics based on the SMART Goal Evaluation Method (SMART‐GEM); evidence‐based strategies and worked examples.^52^ As a consequence of the feedback received by participants on their experience, the frequency of messages per goal is similar to the Phase I trial, but there is the option for participants to select up to five goals. Messages are being sent via SMS or email, depending on personal preference. Additional changes resulting from this Phase I study, include supplementary information provided to participants to improve the accessibility of the embedded weblinks, with further instruction on the importance of checking their spam/junk email for messages. An electronic option is also being offered to complete the satisfaction survey to increase response rates.

One limitation to the results of our study is the small sample for the survey and focus group. We acknowledge that saturation was not able to be determined from just one focus group. However, the purposively selected participants with various views and experiences of the programme provided richness of data, and sufficient information power,^53^ and richness of data. Similar themes also emerged from the survey and focus group responses when results were triangulated. Having an experienced interviewer, with stroke expertize, and emphasizing the importance of both positive and negative feedback from participants in the focus group, minimized the potential of social desirability bias and ensured that a range of assumptions and perspectives were considered. Although the interviewer (T. P.) was involved in goal setting with some participants, those in the focus group were unaware of this involvement, so responses would not be biased. The use of individual study identification numbers meant participants were not able to be readily identified by the researcher (T. P.). We recognize that people who elect to participate in research such as this trial are typically more health conscious than the general population. The potential of a biased sample is also emphasized by the numerous focus group participants who reported setting goals on some level before recruitment to the iVERVE trial. While goal attainment is a valid measure of assessing goal achievement, it does not account for intermediate goals to be explored, and we also did not collect specific information on actions that participants undertook in working towards their goals.

We further acknowledge the divergent views of participants related to aspects of the programme such as the motivational benefit of the messages. However, this early stage research is a step in developing a programme that can be scaled up. Further exploration of participant perceptions and underlying causal effects is required, and these aspects will be of interest in the process evaluation embedded in the future trials of the iVERVE intervention. Lastly, the addition of an online/virtual option for focus group participation may have improved the likelihood of participants being involved. Nevertheless, our results offer important insights that contribute to the current understanding of how people living with stroke engage with digital health programmes and their willingness to use these tools.

## 6 CONCLUSIONS

The participant experience related to the iVERVE programme for this stroke population highlights the potential of using digital health interventions to address self‐management issues faced by people living with stroke. This valuable feedback has informed refinements to our ongoing Phase II and III trials and should be used to shape future research in this field.

## AUTHOR CONTRIBUTIONS

Dominique A. Cadilhac conceived and designed the study, acquired grant funding, drafted the manuscript and revised it for critical content. Nadine E. Andrew, Doreen Busingye, Jan Cameron, Amanda G. Thrift, Natasha A. Lannin, Monique F. Kilkenny and Ian Kneebone were involved in various aspects of the study design and protocol development. Tara Purvis and Doreen Busingye undertook the data collection. Tara Purvis, Dominique A. Cadilhac, Monique F. Kilkenny, Jan Cameron contributed to the design of the statistical analysis approach, data analysis and interpretation. Tara Purvis drafted the manuscript, and all authors critically revised successive drafts of the manuscript for important intellectual content. All authors have read and approved the final version of the manuscript.

## CONFLICT OF INTEREST

The authors declare no conflict of interest.

## Supporting information

Supplementary information.

Supplementary information.

Supplementary information.

Supplementary information.

Supplementary information.

## Data Availability

Data that support the findings of this evaluation are available from the corresponding author upon reasonable request.
