# Supplementary material for: Mixed methods evaluation to explore participant experiences of a pilot randomized trial to facilitate self‐management of people living with stroke: Inspiring virtual enabled resources following vascular events (iVERVE)
Source: Health Expect. 2022 Aug 23;25(5):2570–81. doi: 10.1111/hex.13584 (PMC9615081; doi:10.1111/hex.13584)
Supplement: Supplementary file 1 — Supplementary information. [file HEX-25--s001.docx]

**Reporting Guideline: SURGE (The SUrvey Reporting GuidelinE)**

Grimshaw JM. SURGE (The SUrvey Reporting GuidelinE). In: Moher D, Altman D, Schultz K, Simera I, Wager E, eds. Guidelines for Reporting Health Research: A User's Manual. UK: John Wiley & Sons; 2014

| **Category** | **Item** | **Page** |
| --- | --- | --- |
| Title and abstract | Is the design of the study stated in the title and/or abstract? | Title & abstract |
| Introduction | Is there an explanation of why the research is necessary, placing the study in context of previous work in relevant fields? | Abstract & 3,4 |
|  | Is the purpose or aim of the paper explained? | 4 |
| Methods |  |  |
| Research tool | Is the questionnaire described? | 6 & Supplemental File A |
|  | If an existing tool was used, are its psychometric properties presented? | N/A |
|  | Is an existing tool was used, are references to the original work provided? | N/A |
|  | If a new too was used, are the procedures used to develop and pre-test provided? | 6 |
|  | If a new too was used, have its reliability and validity been reported? | - |
|  | Is a description of the scoring procedures provided? | 6 |
| Sample selection | Is there a description of the survey population and the sample frame used to identify this population? | 6 |
|  | Do the authors provide a description of how representative the sample is of the underlying population? | 7 & Supplemental File C |
|  | Is a sample size calculation or rationale / justification for the sample size presented? | N/A |
| Survey administration | Mode of administration? | 6 |
|  | Do the authors provide information on the type of contact and how many attempts were made to contact subjects (i.e. prenotification by letter or telephone, reminder postcard, duplicate questionnaire with reminder)? | 6 |
|  | Do the authors report whether incentives were provided (financial or other)? | 6 |
|  | Is there a description of who approached potential participants (e.g. identification of who signed the covering letter)? | 6 |
| Analysis | Is the method of data analysis described? | 7,8 |
|  | Do the authors provide methods for analysis of nonresponse error? | N/A |
|  | Is the method for calculating response rates provided? | 8 |
|  | Are definitions provided for complete versus partial completion? | 7 |
|  | Are the methods for handling item missing data provided? | 7 |
| Results | Is the response rate reported? | 8 |
|  | Are all respondents accounted for? | 8 |
|  | Is information given on how nonrespondents differ from respondents? | 8 & Supplemental File C |
|  | Are the results presented clearly? | 8-13, Figures 3-5 |
|  | Do the results address the objective(s)? | As above |
| Discussion | Are the results summarized with reference to the study objectives? | 14-16 |
|  | Are the strengths of the study stated? | 16 |
|  | Are the limitations of the study (taking into account potential sources of bias or imprecision) stated? | 17,18 |
|  | Is there explicit discussion of the generalizability (external validity) of the results? | - |
| Ethical quality indicators | Study funding reported? | 20 |
|  | Research Ethics Board (REB) review reported? | 8 |
|  | Reporting of subject consent procedures? | 6 |

**COREQ (COnsolidated criteria for REporting Qualitative research) Checklist**

Developed from: Tong A, Sainsbury P, Craig J. Consolidated criteria for reporting qualitative research (COREQ): a 32-item checklist for interviews and focus groups. International Journal for Quality in Health Care. 2007. Volume 19, Number 6: pp. 349 – 357

| **No.** | **Item** | **Guide questions/ description** | **Page** |  |
| --- | --- | --- | --- | --- |
| **Domain 1: research team and reflexivity** | | | | |
| Personal Characteristics | | | | |
| 1 | Interviewer/facilitator | Which author/s conducted the interview or focus group? | 7 |  |
| 2 | Credentials | What were the researchers credentials? *E.g. PhD, MD* | 7 |  |
| 3 | Occupation | What was their occupation at the time of the study | 7 |  |
| 4 | Gender | Was the researcher male or female? | 7 |  |
| 5 | Experience and training | What experience or training did the researcher have | 7 |  |
| Relationship with participants | | | |  |
| 6 | Relationship established | Was a relationship established prior to study commencement? | 7,18 |  |
| 7 | Participant knowledge of the interviewer | What did the participants know about the researcher? *E.g. personal goals, reasons for doing the research* | 7,18 |  |
| 8 | Interviewer characteristics | What characteristics were reported about the interviewer/facilitator? *E.g Bias, assumptions, reasons and interests in the research topic* | 7,18 |  |
| **Domain 2: study design** | | | |  |
| Theoretical framework | | | |  |
| 9 | Methodological orientation and theory | What methodological orientation was stated to underpin the study? *E.g. grounded theory, discourse analysis, ethnography, phenomenology, content analysis* | - |  |
| Participant selection | | | |  |
| 10 | Sampling | How were participants selected? *E.g. purposive, convenience, consecutive, snowball* | 6 |  |
| 11 | Methods of approach | How were participants approached? *E.g. face-to-face, telephone, mail, email* | 6 |  |
| 12 | Sample size | How many participants were in the study? | 6 |  |
| 13 | Non-participation | How many people refused to participate or dropped out? Reason? | 7, Figure 2 |  |
| Setting | | | |  |
| 14 | Setting of data collection | Where was the data collected? *E.g. home, clinic, workplace* | 6,7 |  |
| 15 | Presence of non-participants | Was anyone else present besides the participants and researchers? | 7 |  |
| 16 | Description of sample | What are the important characteristics of the sample? *E.g. demographic data, date* | Table 1 |  |
| Data collection | | | |  |
| 17 | Interview guide | Were questions, prompts, guides provided to the authors? Was it pilot tested? | Supplemental File B |  |
| 18 | Repeat interviews | Were repeat interviews carried out? If yes, how many? | No |  |
| 19 | Audio/visual recording | Did the research use audio or visual recording to collect the data? | 7 |  |
| 20 | Field notes | Were field notes made during and/or after the interview of focus group | 7 |  |
| 21 | Duration | What was the duration of the interviews or focus group? | 7 |  |
| 22 | Data saturation | Was data saturation discussed? | 17 |  |
| 23 | Transcripts returned | Were transcripts returned to participants for comment and/or correction? | No |  |
| **Domain 3: analysis and findings** | | | |  |
| Data analysis | | | |  |
| 24 | Number of data coders | How may coders coded the data? | 7 |  |
| 25 | Description of the coding tree | Did authors provide a description of the coding tree? | 7 |  |
| 26 | Derivation of themes | Were themes identified in advance or derived from the data? | 7 |  |
| 27 | Software | What software, if applicable, was used to manage the data? | 7 |  |
| 28 | Participant checking | Did participants provide feedback on the findings? | No |  |
| Reporting | | | |  |
| 29 | Quotations presented | Were participant quotations presented to illustrate the themes/findings? Was each quotation identified? *E.g. participant number* | Throughout results |  |
| 30 | Data and findings consistent | Was there consistency between the data presented and the findings? | Throughout results |  |
| 31 | Clarity of major themes | Were major themes clearly presented in the findings? | Throughout results |  |
| 32 | Clarity of minor themes | Is there a description of diverse cases or discussion of minor themes? | Throughout results |  |
