# Supplementary material for: Mixed methods evaluation to explore participant experiences of a pilot randomized trial to facilitate self‐management of people living with stroke: Inspiring virtual enabled resources following vascular events (iVERVE)
Source: Health Expect. 2022 Aug 23;25(5):2570–81. doi: 10.1111/hex.13584 (PMC9615081; doi:10.1111/hex.13584)
Supplement: Supplementary file 2 — Supplementary information. [file HEX-25--s005.docx]

**iVERVE Satisfaction Survey (control)**

***Thank you for participating in this study. Please tell us your opinion about the goal setting process and overall trial. This information will be helpful in improving the study. Please return the completed form to our office using the pre-addressed postage paid envelope***

| **Participant ID** |  |
| --- | --- |
| **GOAL SETTING**  ***Please indicate by ticking ONE of the boxes which statements you agree with most and provide more details where relevant*** |  |
| **Strongly Agree Neutral Disagree Strongly**  **agree disagree** |  |
| 1. Did you find the goal setting form we provided  helpful in developing your goals? |  |
| 2. Was the health professional helpful in  developing your recovery/health goals? |  |
| 3. Was the goal setting form easy for you to  understand? |  |
| 4. Did you require help in making sense of some  items on the goal setting form? |  |
| 5a. Were there any goals that you wanted to set that were not on the goal setting form?  Yes No |  |
| 5b. If yes, what additional items would you like to have had on the form?  …………………………………………………………………………………………………… |  |
| 6. Do you have any suggestions on how the goal setting form and process could be improved:  Design:…………………………………………………………………………………………………  Content:……………………………………………………………………………………………………  Other:…………………………………………………………………………………………………….. |  |
| **OVERALL STUDY SATISFACTION**  ***Please indicate by ticking ONE of the boxes which statements you agree with most and provide more details where relevant*** | |
| **Strongly Agree Neutral Disagree Strongly**  **agree disagree** | |
| 7. I would be willing to be part of a similar project  in future to help me manage my stroke | |
| 8. The project has helped me set goals that are  reasonable and within reach | |
| 9. I thought the project content was very relevant  to my situation | |
| 10. I trust the information and advice I was given in  the project | |
| 11. I felt it was worth my time and effort to take part  in the project | |
| 12. If available I would recommend this project to  other people with stroke | |
| 13. In your opinion, how could we improve this project overall?  ……………………………………………………………………………………………………….  ……………………………………………………………………………………………………….. | |
| 14. Do you have any other feedback or comments about this project?  ………………………………………………………………………………………………………….  …………………………………………………………………………………………………………. | |

**iVERVE Satisfaction Survey (intervention)**

***Thank you for participating in this study. Please tell us your opinion about the usefulness and acceptability of support messages and the goal setting process. This information will be helpful in improving the study. Please return the completed form to our office using the pre-addressed postage paid envelope***

| **Participant ID** |  |
| --- | --- |
| **GOAL SETTING**  ***Please indicate by ticking ONE of the boxes which statements you agree with most and provide more details where relevant*** |  |
| **Strongly Agree Neutral Disagree Strongly**  **agree disagree** |  |
| 1. Did you find the goal setting form we provided  helpful in developing your goals? |  |
| 2. Was the health professional helpful in  developing your recovery/health goals? |  |
| 3. Was the goal setting form easy for you to  understand? |  |
| 4. Did you require help in making sense of some  items on the goal setting form? |  |
| 5a. Were there any goals that you wanted to set that were not on the goal setting form?  Yes No |  |
| 5b. If yes, what additional items would you like to have had on the form?  …………………………………………………………………………………………………… |  |
| 6. Do you have any suggestions on how the goal setting form and process could be improved:  Design:…………………………………………………………………………………………………  Content:……………………………………………………………………………………………………  Other:…………………………………………………………………………………………………….. |  |
| **ELECTRONIC SUPPORT MESSAGES**  ***Please indicate by ticking ONE of the boxes which statements you agree with most and provide more details where relevant*** |  |
| **ACCEPTABILITY** |  |
| **Strongly Agree Neutral Disagree Strongly**  **agree disagree** |  |
| 1. Using text/email messages is a good way to  teach me about stroke |  |
| 2. I was comfortable in accessing and using my  mobile phone to read and respond to text messages? |  |
| 3. I was able to easily understand the text/email  messages |  |
| 4a. The frequency of text/email messages suited me Yes No |  |
| 4b. If you answered no, what would be your preferred frequency e.g. daily, once/twice a week?  ……………………………………………………………………………………………………….. |  |
| 5a. The text/email messages were sent at appropriate time of the day for me  Yes No |  |
| 5b. If you answered no, how would you have liked your messages timed?  ………………………………………………………………………………………………………….. |  |
| 6a. The number of messages I received each week was just right  Yes No |  |
| 6b. If you answered no, how many messages would you have liked to have received each week?  Comment:………………………………………………………………………………………………… |  |
| 7a. Was the duration/length that the support messages were delivered, sufficient for your needs? Yes No |  |
| 7b. If you answered no, how many weeks would you have liked the electronic messages to continue?  Comment:………………………………………………………………………………………………… |  |
| 8. I liked receiving messages with my name Strongly agree  Agree  Neutral  Disagree  Strongly disagree |  |
| 9. I liked receiving messages with the iVERVE team signature at the end  Strongly agree  Agree  Neutral  Disagree  Strongly disagree |  |
| 10a. I liked reading these electronic messages that were sent to me that refered to:  **Yes No N/A** |  |
| Medications |  |
| Nutritional information |  |
| Physical activity |  |
| Smoking |  |
| Alcohol consumption |  |
| Weight management |  |
| Health and body functions e.g mobility, fatigue, falls, pain, emotions etc. |  |
| Activities and participation e.g relationships, leisure activities, dressing |  |
| Access to information, health professionals and online support |  |
| Motivational support/encouragement to achieve goals |  |
| Sources of additional information |  |
| 10b. If you answered no to any of the above, please give a reason why you did not like the messages………………………………………………………………………………………………….  …………………………………………………………………………………………………………….. |  |
| **BENEFITS (please tick one of the boxes)** |  |
| **Strongly Agree Neutral Disagree Strongly**  **agree disagree** |  |
| 11. I was inspired by the motivational messages |  |
| 12. The recovery goal-specific messages helped  me to achieve my goals |  |
| 13. The messages increased my health  awareness |  |
| 14. The messages helped me to adopt  healthy lifestyle behaviours |  |
| 15. The messages increased my self-management  skills |  |
| 16. It was easy to receive and read the text/email  messages from the research team |  |
| 17. I understood how to access further information  from the web links provided in the messages |  |
| **CONFIDENCE** |  |
| 18. Please indicate below, if you use these electronic devices and how frequently you use each of them. If you answer Yes to Q18a, please answer Q18b |  |
| \| **18a. I use a** \|  \|  \| **18b. I use this electronic device** \| \| \| \| \| --- \| --- \| --- \| --- \| --- \| --- \| --- \| \| Standard mobile phone (e.g. Nokia) \| Yes \|  \| **🡪** \| Daily \|  \| Monthly \| \|  \| \| No \|  \|  \| Weekly \|  \| Rarely \| \|  \| \| N/A \| \| 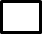 \| Fortnightly \| \|  \| Never \| \|  \| \| Smart phone (e.g. iphone or android) \| Yes \|  \| **🡪** \| Daily \|  \| Monthly \| \|  \| \| No \|  \|  \| Weekly \|  \| Rarely \| \|  \| \| N/A \| \| 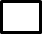 \| Fortnightly \| \|  \| Never \| \|  \| \| Tablet/ipad \| Yes \|  \| **🡪** \| Daily \|  \| Monthly \| \|  \| \| No \|  \|  \| Weekly \|  \| Rarely \| \|  \| \| N/A \| \| 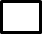 \| Fortnightly \| \|  \| Never \| \|  \| \| **18a. I use a** \| \|  \| **18b. I use this electronic device** \| \| \| \| \|  \| \| Laptop \| Yes \|  \| **🡪** \| Daily \|  \| Monthly \| \|  \| \| No \|  \|  \| Weekly \|  \| Rarely \| \|  \| \| N/A \| \| 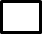 \| Fortnightly \| \|  \| Never \| \|  \| \| Desktop computer \| Yes \|  \| **🡪** \| Daily \|  \| Monthly \| \|  \| \| No \|  \|  \| Weekly \|  \| Rarely \| \|  \| \| N/A \| \|  \| Fortnightly \| \|  \| Never \| \|  \| \| Other (please specify) \| Yes \|  \| **🡪** \| Daily \|  \| Monthly \| \|  \| \| No \|  \|  \| Weekly \|  \| Rarely \| \|  \| \| N/A \| \|  \| Fortnightly \| \|  \| Never \| \|  \| |  |
| 19. Do you need help from another person to use any of the electronic devices selected above?  Yes No | |
| 20. Please indicate the functions of your phone that you are able to use (***check all that apply***)  Make calls using my contact list  Receive calls  Compose and send text messages  Access received text messages  Search the web  Use Apps | |
| 21. Do you use the SMS (text) functions on your phone to send or read messages?  Yes No N/A | |
| 22. I am more confident in using electronic devices to find health information since completing this study. Yes No | |
| **USABILITY** | |
| 23a. After a while I did not read the messages Yes No | |
| 23b. If you answered yes, how many weeks after starting the program did you stop reading the messages?  Weeks | |
| 23c. What are the reasons for you not reading the messages?...................................................  ………………………………………………………………………………………………………………  ……………………………………………………………………………………………………………. | |
| 24. Please indicate which times of day you would have preferred to receive text/email messages (***check all that apply***)  Morning  Afternoon   Evening (6pm-8pm)  Any time | |
| **OVERALL STUDY SATISFACTION**  ***Please indicate by ticking ONE of the boxes which statements you agree with most and provide more details where relevant*** | |
| **Strongly Agree Neutral Disagree Strongly**  **agree disagree** | |
| 25. I would be willing to be part of a similar project  in future to help me manage my stroke | |
| 26. The project has helped me set goals that are  reasonable and within reach | |
| 27. I thought the project content was very relevant  to my situation | |
| 28. I trust the information and advice I was given in  the project | |
| 29. I felt it was worth my time and effort to take part  in the project | |
| 30. If available I would recommend this project to  other people with stroke | |
| 31. In your opinion, how could we improve this project overall?  ……………………………………………………………………………………………………….  ……………………………………………………………………………………………………….. | |
| 32. Do you have any other feedback or comments about this project?  ………………………………………………………………………………………………………….  …………………………………………………………………………………………………………. | |
