# Supplementary material for: Mixed methods evaluation to explore participant experiences of a pilot randomized trial to facilitate self‐management of people living with stroke: Inspiring virtual enabled resources following vascular events (iVERVE)
Source: Health Expect. 2022 Aug 23;25(5):2570–81. doi: 10.1111/hex.13584 (PMC9615081; doi:10.1111/hex.13584)
Supplement: Supplementary file 3 — Supplementary information. [file HEX-25--s003.docx]

**iVERVE Project Focus Group discussion questions**

***Focus group facilitator to read out****: Thank you for participating in the iVERVE project. The aim of this part of the evaluation is to obtain further information about the impact of the project on you, the stroke survivors who were involved. In particular we would like to hear your views about the goal setting process and also the electronic support including the text and email messages and whether these were useful.*

*It is important that we get this feedback from your perspective, regardless of whether it is positive or negative because we want to improve our work and ensure it is relevant.*

*Interviews will be recorded and then transcribed, and an overall summary will be presented based on the various themes & information identified. In any reporting of this information, you will not be identified by name. Specific quotes may distinguish between a male or female respondent e.g. F1, M1, F2 etc.*

**Goal setting form and process of setting goals**

***Focus group facilitator to read out****: At the start of the study, we sent you a form in the mail to help you set your health or recovery goals [hold up paper copy as a reminder]. A health professional then assisted you to set specific and meaningful goals over the phone. We would like to find out your thoughts about this goal setting process.*

1. Do you remember receiving the goal setting form?

a) Did you look at it- why, why not?

b) Was it easy to understand or not? What did you find difficult to understand?

- Please suggest how we could have made it easier for you to understand.

c) Was it helpful in setting goals for yourself?

d) Did the goal setting form cover all the goals that you wanted?

- What additional items would you like to have had on the goal setting form?

e) Do you have any additional suggestions for how the goal setting form could be improved or made more useful?

2. Can you describe your experience with the actual goal setting process with the health professional…

Prompt questions:

1. Did you find the assistance provided by the health professional helpful?
2. Please explain why the process of setting goals was helpful or not? Why/why not
3. Did you feel that you directed the goal setting?
4. Did you feel that the goals set were realistic and meaningful to you, and achievable?
5. Do you think setting these goals has influenced your life after stroke and recovery?

3. Do you remember receiving a written copy of the goals set with the health professional?

Was this useful/not?

**Electronic Support**

1. How did you find, or what was your experience with, the electronic support- which included the text messages or emails in supporting you achieving your goals and providing information and support after stroke?

Prompt questions:

a) Do you believe the messages were useful? Did you feel they were related to your health goals that you set?

b) Why was it useful or why was it not useful?

c) What aspects did you like?

d) What did you not like?

e) What would you want improved about this electronic support?

2. With respect to the content of the messages, did you find these:

Prompt questions:

a) Easy to understand or difficult?

b) What was difficult to understand?

3. Which types of messages e.g. medications, motivational were most or least useful?

4. How did you find the delivery of the electronic messages?

a) Frequency

b) Timing

c) Number of messages per week

5. How did you find the duration/length of the electronic support?

Prompt questions:

a) Was it sufficient or not?

b) What duration would you consider sufficient and why?

6. Are there any drawbacks or limitations you experienced in using or accessing this electronic support? (prompt technical)

7. Do you have any other additional suggestions for how this electronic support could be improved?

**We realised that relevance from time of stroke is important. We plan to provide this to new patients who leave hospital after stroke once they are about two weeks into being back at home.**

**Given this context, do you think this program would be worthwhile?**

**Would it mean you have more goals to address?**

**We offered this trial program to you for 4 weeks. How long do you think the program of electronic support should be provided? (prompt again regarding for new stroke)**

**General question**

1. In your opinion how can we improve this project overall?
