# Supplementary material for: Mixed methods evaluation to explore participant experiences of a pilot randomized trial to facilitate self‐management of people living with stroke: Inspiring virtual enabled resources following vascular events (iVERVE)
Source: Health Expect. 2022 Aug 23;25(5):2570–81. doi: 10.1111/hex.13584 (PMC9615081; doi:10.1111/hex.13584)
Supplement: Supplementary file 4 — Supplementary information. [file HEX-25--s002.docx]

*Table: Characteristics of survey responders and non-responders*

|  | Responders  n/N (%), *N=27* | Non-responders  n/N (%), *N=27* |
| --- | --- | --- |
| Sex, male | 16/27 (59) | 17/27 (63) |
| Age, median (Q1, Q3) | 70 (59, 78) | 70 (64, 76) |
| Married, with partner | 15/27 (56) | 17/27 (65) |
| Lived alone | 9/27 (35) | 7/27 (26) |
| Lived home or unit | 25/25 (100) | 26/26 (100) |
| Employment status |  |  |
| Employed/Volunteer* | 9/25 (36) | 10/26 (38) |
| Unemployed | - | 1/26 (4) |
| Retired | 16/25 (64) | 15/26 (58) |
| Preference for electronic messages  SMS  Email | 12/27 (44)  15/27 (56) | 14/27 (52)  13/27 (48) |
| Number of goals set, median (Q1, Q3) | 2 (2, 3) | 2 (1, 3) |
| Major categories of goals set |  |  |
| Secondary prevention | 19/59 (32) | 23/53 (43) |
| Health/body function | 17/59 (29) | 12/53 (23) |
| Activities and participation | 16/59 (27) | 17/53 (32) |
| Environment | 7/59 (12) | 1/53 (2) |

Q1: 1^st^ quartile; Q3: 3^rd^ quartile; SMS: short message service; *full, part time or casual employment, or volunteer work
