# Supplementary material for: Mixed methods evaluation to explore participant experiences of a pilot randomized trial to facilitate self‐management of people living with stroke: Inspiring virtual enabled resources following vascular events (iVERVE)
Source: Health Expect. 2022 Aug 23;25(5):2570–81. doi: 10.1111/hex.13584 (PMC9615081; doi:10.1111/hex.13584)
Supplement: Supplementary file 5 — Supplementary information. [file HEX-25--s004.docx]

**Examples of participant stated goals and finalised SMART goals**

| **Goal menu category** | **Participant stated goal** | **SMART goal** |
| --- | --- | --- |
| Activities and participation | I want to volunteer at the local nursing home | By week 4, to spend at least 2 hours, once per week volunteering at Mayflower Aged Care Facility in Reservoir. |
| Activities and participation | Get out at night more often to see friends | On a Friday/Saturday evening once/fortnight, to get ‘out of the house’ to catch up with friends for a minimum of 2 hours |
| Secondary prevention | To lose some weight | In the next 4 weeks, to lose 2 kg of weight, as measured on the home scales, in the morning before breakfast, with no clothes on |
| Health and body function | Improve my writing | To improve legibility of ‘running’ or cursive writing to 70% of pre-stroke, as determined by self-report, when writing 5 sentences of > 6 words using a pen. |
| Activities and participation | Go away in the caravan more often | To attach and tow the caravan to Swan Hill, set it up, stay for at least 5 nights, and tow back home. To travel with my wife, but I will do all the driving and dealings with the caravan |
| Secondary prevention | To do my own routine of filling and managing medication each week | Once per week, to be able to accurately fill the dosette box* with ALL required medication for the week without missing any tablets, and to identify if there are not enough tablets for the need week and contact the doctor to organise a new script |
| Health and body function | To walk more | To walk on a treadmill in the garage, 4 times per week, with no incline, on a speed of 4km/hr, for at least 15min each time without stopping |
| Environment | To feel safer at home | To meet with general practitioner at least once in 4 weeks, to discuss options and strategies to alert friends/family/relevant authorities if something happens to me when I am home alone |
| Environment | Get more information about pre-diabetic diet | Access information regarding healthy diet specifically related to pre-diabetes. This will be done by accessing the EnableMe website once over the next 4 weeks |
| Activities and participation | Get back to line-dancing | Attend line dancing in Village Community Centre- 1 hour class twice in the next 4 weeks. To only do movements that I’m comfortable with and avoid any twisting movements |
| Secondary prevention | Do home exercise program set by physio at XX Health | Complete home exercise program as prescribed by physio at XX Health once/day, everyday for the next 4 weeks |
| Health and body function | To improve my urinary incontinence | To be able to make it to the toilet 23 out of 4 times without a urinary accident during the day, and only have a change of pad due to soiling twice/day |
| Health and body function | To remain more focussed and concentrated in meetings at work | To improve self-reported focus and concentration during late morning meetings at work to 8/10 (average over the entire week) |

***tablet/medication organiser**
